# Mathematical Methods in Biomedical Imaging

**DOI:** 10.1155/2013/932794

**Published:** 2013-12-10

**Authors:** Wenxiang Cong, Kumar Durairaj, Peng Feng

**Affiliations:** ^1^Biomedical Imaging Center, Department of Biomedical Engineering, Rensselaer Polytechnic Institute, Troy, NY 12180, USA; ^2^Department of Electrical and Electronics Engineering, Periyar Maniammai University, Vallam, Thanjavur 613403, India; ^3^Department of Optoelectronics Engineering, Chongqing University, Chongqing 400044, China

Biomedical imaging is a rapidly growing field to provide a state-of-the-art tool for preclinical biomedical research and clinical applications, in view of its ability to noninvasively reveal subtle structural variations of biological tissues and visualize in vivo physiological and pathological processes at the cellular and molecular levels. Mathematical methods are involved with imaging theories, models, and reconstruction algorithms in biomedical imaging. X-ray computed tomography (CT) was a successful application of mathematical method in medical imaging. The CT mathematical model can be reduced to a Radon transform. The inverse transform of Radon transform is invented by Radon in 1917. Magnetic resonance imaging (MRI) is a versatile medical imaging modality. MRI can provide more diagnostic information than any of the existing imaging techniques. It does not involve the use of ionizing radiation, hence free from associated harmful effects. Inverse Fast Fourier Transform (IFFT) is a standard method of image reconstruction in MRI from uniformly sampled K-space data. From nonuniform K-space data, iterative algorithms can improve image quality of image reconstruction for MRI. In the optical molecular imaging, the radiative transport equation (RTE) is the fundamental equation to describe photon propagation in biological tissues. The forward solution predicts photon propagation in the optical molecular imaging. The inverse solution can reconstruct molecular probe distribution in a small animal for providing unique insights into disease pathogenesis, drug development, and responses of therapy. The solutions for RTE usually involve analytical methods, Monte Carlo (MC) method, diffusion approximation (DA) method, simplified spherical harmonics method, and some numerical methods.

In this special issue, each paper was reviewed by at least two reviewers and revised according to review comments. This special issue covered most of common biomedical imaging models and various image processing methods, such as registration, segmentation, and so forth, were involved. For Positron Emission Tomography (PET) imaging model, two attenuation correction methods based on X-rays CT (CTAC method) and segmentation of emission images (SE-AC method) were simulated with Monte Carlo method and compared. For synchrotron Micro-CT imaging model, a semiautomatic segmentation algorithm for extracting the complete structure of acini has been proposed. For ultrasound imaging model, a common carotid artery segmentation scheme based on active shape model can get better result and promote the translation of carotid 3D US to clinical care for the monitoring of the atherosclerotic disease progression and regression. For MRI model, a mesh-deformation constraints based image registration algorithm was carefully investigated. Also the development of image segmentation for intracranial aneurysms and rotation covariant image processing method for biomedical applications are summarized. Furthermore, in order to accelerate the parallel imaging, a sparse constrained reconstruction approach with variable splitting methods was proposed and verified: total variation (TV)-minimization interior tomography algorithm, dynamic and robust blind watermarking scheme to resist against common distortions, modified global and modified linear contrast stretching techniques for identification of various stages and species of malaria, a 3D surface-based deformable model as guidance for nonrigid 3D medical image registration and fusion, mathematical or computational modeling for oncogene inactivation, a group factor analysis model for neuroimaging applications by assigning separate factor patterns to control and patient groups yielding more reasonable factor scores and patterns, different MISO Volterra methods to model simulated ultrasound contrast agents signals, an automated process that determines whether an aortic object in a slice is a candidate for aortic dissection or PAU based on contrast enhanced CT data, image-based computational techniques to quantify the severity and directionality of individual scratches and scrapes, combination of genetic algorithm and closed loop to obtain optimal ternary command which maximized the contrast to tissue ratio, and magnetoacoustic tomography with magnetic induction (MAT-MI) for generating electrical conductivity images of biological tissues with high spatial resolution. An acceleration strategy for fluorescence molecular tomography (FMT) with early photons is proposed using graphics processing units (GPUs). The fluorescence molecular tomography (FMT) with early photons can efficiently improve the spatial resolution and fidelity of the reconstructed results. An efficient compressed sensing-based algorithm is proposed for CT image reconstruction from few-view data to suppress the streak artifact. The compressed sensing (CS) algorithm shows the potential to accurately recover images from highly undersampled data. The discriminant analysis techniques are discussed using MRI to identify the correlative pattern of brain changes for differentiating parkinsonian syndromes. A hybrid multiscale and multilevel image fusion algorithm for green fluorescent protein (GFP) image and phase contrast image of Arabidopsis cell is proposed. This algorithm uses different fusion strategies for different detailed subbands, which include neighborhood consistency measurement (NCM) that can adaptively find balance between color background and gray structure. A detrended fluctuation analysis (DFA) method is applied to image analysis to investigate the characteristic of different type of simulated and lymphoma image. A method is presented to estimate the tilt and decentration of intraocular lens (IOL) more accurately. The Bayesian hierarchical model for the analysis of categorical longitudinal data is investigated from sedation measurement for MRI and CT. In vivo MRI of local drug delivery is discussed to visualize and quantify the time resolved distribution of MRI contrast agents.

These papers represent an insightful observation into the state of the art, as well as future topics in this biomedical imaging field. We hope that this special issue would attract a wide attention of the peers.

*Wenxiang Cong* *Kumar Durairaj* *Peng Feng*

